# 30 years of the transformation of non-urban public transport in Poland’s peripheral areas — what went wrong?

**DOI:** 10.1007/s11629-021-6762-y

**Published:** 2021-11-18

**Authors:** Ariel Ciechański

**Affiliations:** Stanisław Leszczycki Institute of Geography and Spatial Organization, Polish Academy of Sciences, 00-818 Warsaw, Poland

**Keywords:** Carpathians, Public transport, Transport networks, Transport-based social exclusion, Poland

## Abstract

Transport-based social exclusion is currently a serious social problem in Poland, and one which is apparently most severe at the level of the county (Polish *powiat*) in the south-east of the country, including the Beskid Niski and Bieszczady Mountains. A deeper illustration of this problem requires both observation of changes in the suburban public-transport network and the identification of areas in which this has deteriorated significantly in quantity and quality. The chosen starting point for the research was therefore 1990, as a year in which — on the one hand — the Polish economy was already shifted to the new free-market principles; while — on the other — state PKS (Przedsiębiorstwo Komunikacji Samochodowej) non-urban bus transport enterprises still dominated public transport. The endpoint of the study is then the beginning of 2019 (the author’s research year). The article introduced here seeks to identify and present cartographically the changes affecting the public-transport network in the study area over the last 30 years, as well as to point to possible consequences of these processes. The background of the described changes is also discussed, as are the observed consequences of what is taking place.

## 1 Introduction

Knowles (1993) noted that the major research challenges of transport geography in the 1990s would include the problem of the growing gap between the mobility of the poor (or other disadvantaged groups) and the rich; as well as inequalities in the meeting of transport needs. Among other things, this reflects a shift on the labour market, where it is possible to observe reduced numbers of jobs and services in rural areas, with positions and services moving to suburban locations with access to roads. Lucas (2019) confirms that transport-based social exclusion has remained an important and up-to-the-minute research problem. She also notes how – for more than 15 years now – this has been among key issues tackled in the *Journal of Transport Geography*, with scientific achievements in this area seen to be expanding constantly.

Church et al. (2000) proposed the more precisely-defined forms of transport-based social exclusion which we address in our study. The Beskid Niski and Bieszczady Mountains of Poland are uplands of a peripheral character linked only by poor transport connections and with a lack of potential destinations, or else a distance from the latter that discourages people from travelling (through geographical exclusion and exclusion from facilities). Additionally, the nature of transport systems may cause physical and/or psychological difficulties which stop potential users (via physical exclusion), while the time needed for care, work, self-fulfilment and other commitments may limit travel possibilities and/or limit people to travelling freely in those moments when fewer transport services are available (via time exclusion).

The network of public transport beyond towns and cities is in a constant state of evolution. However, while ongoing changes in the connections offered by passenger rail transport are easy to document, the same cannot be said (these days) when it comes to coach and bus services operating in the countryside (Ciechański 2019). For this reason the main aim of the work underpinning this article is to identify and present cartographically the changes in the public transport network in the study area taking place over the last 30 years, as well as to indicate the possible consequences of these processes. One such consequence may be the growing problem of transport-based social exclusion. The main subject of the research is primarily non-urban public transport and the changes in the network thereof. Based on its definition from Poland's *Act on Mass Public Transport* (*Ustawa o publicznym transporcie zbiorowym* 2011), it can be assumed that this is widely available and regular passenger transport made available at specific intervals and along a specific line or transport network, for which the organiser is not a city government.

The author adopts the hypothesis that peripheral areas have been particularly vulnerable to the negative changes in the public-transport network arising out of 30 years of Poland's political and economic transformation. It was for this reason that the area of the Beskid Niski and Bieszczady Mountains was chosen as the case study. This is a very specific part of Poland with significant potential in tourism, but at the same time being of a very peripheral nature that geopolitical factors have further deepened in the years since 1945. An additional reason for undertaking the research in this area was the fact that recent years have brought particular impacts due to cuts in the network of transport connections resulting from the liquidation of most of the former state-owned PKS (*Przedsiębiorstwo Komunikacji Samochodowej*) coach-transport companies (Taylor and Ciechański 2020). This left the area particularly vulnerable to the processes of transport-based social exclusion.

The issues this article deals with fall within several different strands of geographical research. While the main sphere is obviously transport geography, there is also at least tangential involvement of matters relating to border areas and peripheries. Relying on the dichotomous division arrived at by Friedmann (1967) – in which cores contrast with peripheries – it is definitely the latter type of area that the studied region of the Beskid Niski and Bieszczady Mountains embodies. Further reference might be made to the definition from Goodall (1987, p 350), holding that these are areas both distant from the centres of economic growth and difficult of access for transport-based social reasons. The peripheral status is undoubtedly enhanced by the borderland location, which confers upon the study areas peripherality of a so-called genetic origin (Grosse 2007). Furthermore, returning to the division offered by Friedmann and Alonoso (1964), we would find the study area also conforming to the definition of marginal region and region that is run-down and/or lagging behind.

The work presented here has thus sought to identify changes in public transport away from the major towns and cities of Poland's Beskid Niski and Bieszczady region, and to point to possible consequences of relevant processes taking place there. The significance of the research problem is emphasised by the fact that the processes in question that are to be observed (regression of the public-transport network and increased transport-based social exclusion) will go on growing. For this reason, it is necessary to diagnose the current situation.

Social exclusion is defined as a lack of participation by certain people in social, economic and political life. It thus has a broader meaning than poverty, and is multidimensional and cumulative. Of course, limited financial resources and security are often interrelated with a low level of education and skills, poor health, limited political strength, etc. Social exclusion is also relative to other people or groups, dynamic, subject to change over time and multidimensional in that it is experienced by individuals who are affected directly – as are their households, neighbourhoods and local communities (Schwanen et al. 2015). On the other hand, official documents of the EU indicate that social exclusion is a condition restricting access to the labour market, healthcare services, education, etc. – for individuals, groups or communities. It results in an inability of people to participate in many aspects of life typical for the general public (Mężyk 2013).

The process directly connected with the term above is transport-based social exclusion, i.e. social exclusion of a person or group due to the non-availability of transport (Jaroš 2017). A related concept is transport disadvantage – a relative and dynamic result of a lack of access to basic resources, activities and interaction possibilities, as well as cognitive skills, know-how, aspirations and/or autonomy in travel and the associated externalities; as well as their impact on the decision-making process in the context of transport policy and management. Inconveniences in transport can be both absolute and relative, and occur at both the individual and collective levels (Schwanen et al. 2015). Delbosc and Currie (2011) point out that transport disadvantage is recognised generally as a multidimensional concept of characteristics linked closely with location, access to mobility, and access restrictions arising out of a person's mental, social and psychological characteristics. While in the opinion of Stanley et al. (2019), the phenomenon occurs when people suffer from a lack of transport capabilities, it should be borne in mind that these authors make social exclusion more dependent on the narrowly-defined minimum-income criterion, even as they put less emphasis on other component aspects of the concept.

The subject of transport-based social exclusion has long attracted the attention of researchers from many countries. One of the key (holistic) studies is a compendium of knowledge edited by outstanding specialist on this subject (Lucas 2004). The work edited by Currie (2011) is similarly review-like in nature. A separate group of studies are comprehensive reviews of literature and methods regarding transport-based social exclusion or transport disadvantage and the relationship with social exclusion (e.g. Gaffron et al. 2001; Dodson et al. 2004; Dodson et al. 2006; Lucas 2012; Mackett and Thoreau 2015; Kamruzzaman et al. 2016; Jaroš 2017; Lucas 2019). Undoubtedly also of great importance from the point of view of this article's main topic are the studies focusing specifically on transport-based social exclusion in rural areas of Serbia (Ranković-Plazinić and Jović 2018), Northern Ireland (Kamruzzaman and Hine 2011) and Scotland (Velaga et al. 2012).

However, Polish scientific literature on transport-based social exclusion is insufficient. More popular are related topics like mobility and the accessibility of services in rural areas in Poland (e.g. Taylor 1999, 2003; Stępniak et al. 2017) or social consequences of closures of rail connections (Taylor 2006; Karamuz 2011). Only Zmuda-Trzebiatowski (2016) and Kaczorowski (2019) offer some remarks on transport-based social exclusion. More often the Polish literature has focused on the shape of – and changes in – the non-urban public transport network – mainly rail (e.g. Lijewski 1959; Lijewski and Koziarski 1995; Taylor 2007); but less often also the non-urban bus (e.g. Lijewski et al. 1967; Kozanecka 1980; Wolański et al. 2016).

The research area is also a border area. Cavallaro and Dianin (2019) and Medeiros et al. (2021) offer a broader background to cross-border commuting in such areas. The problems of public transport on the Polish borderland are discussed mainly for the Polish-Slovak border (e.g. Więckowski et al. 2012; Michniak and Więckowski 2021), or else the border with the Czech Republic/Czechia (e.g. Kołodziejczyk 2017a, 2017b, 2020).

Still missing (not only in Poland) are holistic, exhaustive investigations into changes in the configuration of public transport connections. At the same time, the last few years have brought a dramatic and far-reaching polarisation of Poland's system of non-urban public bus transport. On the one hand, main routes – e.g. those linking the seats of counties (known as *powiaty* in Polish or "poviats" in English) are being served by more runs than they were before. But on the other hand, areas with low-density populations have experienced a collapse of bus services.

## 2 Method and Materials

It was against the above background that the author took up the challenge of presenting changes in public transport taking place between 1990 and 2019 in the Polish county-level units of administration making up the contiguous hilly and mountainous region in the far south-east of Poland known as the Beskid Niski and the Bieszczady Mountains.

(1) In its first step, the adopted research procedure included the definition of the research area. The criterion of potential special exposure to transport-based processes of social exclusion was adopted here. This factor could have reflected the collapse of, or economic problems with, the local formerly-state-owned PKS transport companies.

(2) The next step was to select the best-fit points on the timeline for more-detailed analysis. While the choice of end date of the study was not problematic – the study year 2019 was adopted, things proved more difficult in the case of the start date. On the one hand, the criterion of the organisation of the network of connections from the communist era was guiding here, but on the other there was a premise regarding the initial stage of functioning of a market economy in Poland. Together, these considerations led to the adoption of a 1990 start date. At the same time, due to the lack of availability of data, intermediate dates were not taken into account.

(3) In seeking to understand better the processes shaping the Polish system of public transport beyond cities, by reference to a dynamic conceptualisation, it was necessary to create a base of timetables encompassing both train and bus/coach travel (for 1990 and 2019).

(4) The collected timetables for 1990 and 2019 provided the basis for the development of databases including information on numbers of connections made on weekdays and restrictions on running that characterised public and school holidays. It was in turn passenger trains that were taken account of as databases for rail transport were constructed. In the case of bus/coach transport, considerations related to regular and express courses (with stops in all towns, but not at all stops).

(5) The next stage of the study was to calculate the difference between the number of pairs of courses for the adopted time sections. This prompted the calculation of the change in the number of pairs of courses on school days in 2019 as compared with 1990.

(6) Once such databases were in hand, it was then possible to draw maps and ribbon diagram cards, before engaging in cartographic analysis to help identify areas most affected by transport exclusion during 30 years of Polish economic and political transformation.

(7) These materials, together with analysis of the literature and Polish legal and strategic acts, were used to draw conclusions and arrive at generalisations regarding analysed processes that point to a growing problem with diminishing educational and social opportunities, primarily among young people and others who cannot or do not use a car.

At the beginning of 1990, the only source of information about changes in the network of public collective transport connections took the form of nationwide book timetables published by *Polskie Koleje Państwowe* (PKP) (for rail carriers) and by *Przedsiębiorstwo Państwowej Komunikacji Samochodowej* (PPKS) (for non-urban bus/coach transport). Set against that background, the situation as of early 2019 can be said to reflect a worsening where access to these sources of information was concerned. While it is true that the railways have continued to come out with definitive timetables, the coach-transport system gradually came to abandon that practice altogether, with the result that a cohesive system of information on out-of-town connections has ceased to exist. In the current situation, the surest source of information is – perhaps paradoxically – the timetable that constitutes an annex to each application for a permit to engage in the regular carriage of passengers – which has to be submitted to local and regional authorities. In the face of a lack of any full and reliable base of timetables, it became necessary to use other sources.

## 3 Case Study Area of the Beskid Niski and Bieszczady Mountains

The research area is located in the south-east of Poland, in mountainous areas of five counties of Podkarpackie Voivodeship (Bieszczady, Lesko, Sanok, Krosno and Jasło) and one county of Małopolskie Voivodeship (Gorlice) – where voivodeships are to be recognised as higher-tier units of organisation in Poland that in fact operate both regional and provincial administrations within the same borders.

These have always been peripheral areas – also located on the Polish-Czechoslovak border in the years 1918(1920)–1938(1939) (Demel 2016). Political change post-1945 only deepened the processes, though the eastern border of the country itself was moved closer to the Polish capital. As a result of changes in the border, the area studied has become even more peripheral, borderland and distant from the natural supra-regional centre that was the former Lvov (currently Lviv on the territory of Ukraine). Until the early 1990s, this was the region of the Polish-Czechoslovak-Soviet border – as opposed to the current situation in which it is Poland, Slovakia and Ukraine that meet here. The periphery of the administrative units constituting the research area was also deepened by the relocation of people of Ruthenian origin. The Beskid Niski were inhabited by the Lemko people, and the Bieszczady by the Boyko (Wolski 2016a). The authorities of communist Poland displaced these ethnic groups in the late 1940s – partly across the new border into the Soviet Union, and partly also to other regions of Poland. A significant part of the villages once inhabited by them have never been re-inhabited, or at best remain settled to a much lesser degree than before the Second World War (Motyka and Pisuliński 2016; Krukar 2016; Wolski 2016b). This state of affairs was additionally affected by the restrictions of the then Polish authorities limiting possibilities for settlement in border areas. All these processes from different points in history continue to affect the demographic situation of these areas through to the present day.

Under the division of Poland into natural regions, what are involved here are the medium-height mountains known as the Beskid Niski (highest peak at 997 m a.s.l.) and the Bieszczady (highest peak at 1346 m a.s.l.) (Kondracki 2000). A small part of the Beskid Niski and a large part of the Bieszczady Mountains are located within the boundaries of National Parks (Więckowski 2018).

The primary subjects of the research detailed here have been rural areas, especially those located deeper into the mountains. On this basis, a group of 12 villages with exceptionally poor transport services or even lacking these altogether was used to measure distances to administrative centres (Table 1). Where physical distance and travel times by private car are concerned, the peripherality in these cases is particularly noticeable, and all the more so when account is taken of the distance to the capital of Poland as a whole – i.e. Warsaw. Equally, even the major provincial cities (regional centres) of Rzeszów (in Podkarpackie Voivodship) and Kraków (in Małopolskie Voivodship) are seen to be separated by considerable distances in both the physical sense and when it comes to time needed to reach them. A better situation applies when account is taken of the tier of the county town or city, but in the case of Lesko and Bieszczady counties it is the role of Sanok that is involved. Although the towns under analysis are relatively close to some local centres (Lesko and Ustrzyki Dolne), the rank of these localities – and thus the range of services (especially higher-order) – available is much more limited than in other county capitals analysed. That leaves the aforementioned Sanok as an important centre and travel destination for many inhabitants of Lesko and Bieszczady counties, this being the largest (if more-distant) county seat in this part of the voivodeship.

**Table 1** Average distances of selected peripheral rural centres from the capitals of their poviats (county) and voivodeships (province), as well as Poland's national capital Warsaw (capital)

| Town | Poviat | Voivodeship | Distance to Poviat | | | Distance to Voivodeship | | | Distance to Warsaw | |
|---|---|---|---|---|---|---|---|---|---|---|
| | | | km | Time(h) | | km | Time(h) | | km | Time by car (h) |
| | | | | by car | by bus/train* | | by car | by bus/train* | | |
| Regietów | Gorlice | Małopolskie | 26 | 0.30 | –(–) | 144 | 2.30 | –(–) | 390 | 5.10 |
| Banica | | | 30 | 0.35 | 0.50(–) | 135 | 2.30 | –(4.30) | 390 | 5.05 |
| Polany | Jasło | Podkarpackie | 39 | 0.40 | 1.00(–) | 87 | 1.30 | –(3.05) | 390 | 5.10 |
| Świątkowa Wielka | | | 35 | 0.45 | 1.10(–) | 99 | 1.45 | –(3.30) | 390 | 5.10 |
| Jaśliska | Krosno | | 33 | 0.35 | 1.00(–) | 85 | 1.30 | –(2.45) | 394 | 5.15 |
| Zyndranowa | | | 34 | 0.35 | –(–) | 88 | 1.30 | –(–) | 393 | 5.15 |
| Nowy Łupków | Sanok | | 47 | 0.50 | –(2.10) | 117 | 2.00 | –(6.00) | 432 | 5.45 |
| Wisłok Wielki | | | 48 | 0.50 | 1.30(–) | 99 | 1.45 | –(5.00) | 411 | 5.25 |
| Wetlina | Lesko | | 57 | 1.00 | 1.25(–) | 144 | 2.30 | –(4.50) | 464 | 6.25 |
| | | | 70 | 1.15 | 1.50(Sanok) | | | | | |
| Wołkowyja | | | 36 | 0.35 | 0.45(–) | 122 | 2.05 | –(3.55) | 442 | 6.00 |
| | | | 48 | 0.50 | 1.10(Sanok) | | | | | |
| Ustrzyki Górne | Bieszczady | | 45 | 0.40 | 1.15(–) | 150 | 2.30 | –(5.10) | 464 | 6.15 |
| | | | 81 | 1.25 | 2.25(Sanok) | | | | | |
| Polana | | | 26 | 0.25 | 0.45(–) | 131 | 2.15 | –(4.20) | 445 | 6.00 |
| | | | 58 | 1.10 | 2.20(Sanok) | | | | | |

**Notes:** Author's own work based on maps.google.com and publicly-available bus timetables.
*Data before the brackets () are the time via direct access and the data in the brackets () are the time via non-direct access by bus/train.

People who do not have access to a private car are in a much less-favourable situation here. The peripheral areas mentioned in the article are marginalised in terms of a public-transport offer that usually comprises less than five pairs of buses a day. An enhancement of the sense of remoteness is also fostered by coach-travel times that are much longer than those involving a private car. There are usually no problems in the case of travel to the nearest county centres, though there are localities (Table 1) from which interchanges are necessary, or which are completely deprived of access to public transport. Interestingly, the aforementioned towns from Lesko and Bieszczady counties were found to have direct connections with Sanok, but the travel times involved here are much longer than in the case of a private car. The situation of the discussed regions is even worse in relation to voivodeship centres (direct access is practically non-existent and transfer with interchanges involves travel times much longer than in the case of a private car, and often even with three or four changes and stopovers).

Jasło, Krosno and Sanok counties are dominated by the chemical industry (including oil-refining). Bieszczady and Lesko counties are of a more agricultural nature, with a still-significant role for the wood-processing industry (Janusz 2016). The peripheral nature of the studied area is also emphasised by economic and social characteristics worse than average for the country as a whole (Table 2). These characteristics become more and more unfavourable at successively-greater distances from the centre of the country. It should be remembered that Krosno county differs from the other five counties in the way that its capital, the city of Krosno, is a separate unit of this type (a so-called "powiat grodzki", which could be translated into English as an urban county or county enjoying town rights). This is also true in terms of the descriptive statistics. These data are not included in Table 2 below, which shows only the so-called "land counties" (*powiaty ziemskie* in Polish). This has major repercussions as, for example in the case of Krosno, it means that data are presented for the Krosno area/region even as those relating to the city itself are excluded.

While in the case of population density as of 2019, Gorlice, Jasło and Krosno counties all display characteristics similar to the national-average values, where Sanok county is concerned these are much less favourable; while for Lesko and Bieszczady counties they are several times lower than the average in Poland. In all six counties, the average salary is markedly below the national average. The distance from the main centres also affects the labour market adversely – in most of the analysed counties the unemployment rate is higher than the Polish average (almost twice as high in the case of Sanok county, and almost three times as high where the counties of Lesko and Bieszczady are concerned). No wonder, then, that all six counties have been depopulating steadily.

Away from any urban centres, this area is of an agricultural character, as supplemented by a tourist function in certain hilly and mountainous parts. The main tourist centres in the Bieszczady Mountains are the towns of Cisna, Wetlina and Ustrzyki Górne more associated with hiking, and the area of the Solina Reservoir more associated with water sports and recreation in the classical understanding of the term. Due to the sparser nature of the network of marked trails and the accommodation base, the Beskid Niski mainly support tourism based around long-distance hiking. The number of hotels and guesthouses is also much smaller here than in the neighbouring Bieszczady Mountains (Więckowski et al. 2012).

## 4 Results – have 30 years of the Polish transformation led to a collapse of non-urban public transport in less-populated areas?

The year 1989 ushered in diametric changes for the whole of Poland and its society. What was in essence set in motion then was an arduous process of transformation from a centrally-planned command

**Table 2** Selected socio-economic characteristics of the poviats (counties) analysed as of 2019

| Poviat | Gorlice | Jasło | Krosno | Sanok | Lesko | Bieszczady | POLAND |
|---|---|---|---|---|---|---|---|
| Average population density [people per km²] | 113 | 137 | 113 | 82 | 32 | 19 | 123 |
| Average gross salary compared with the national average [%] | 74.9 | 79.5 | 73.2 | 80.3 | 85.9 | 82.4 | 100 |
| Unemployment rate [%] | 4.6 | 9.4 | 5.1 | 6.6 | 16.0 | 13.5 | 5.2 |
| Internal migration balance | -300 | -425 | -264 | -414 | -84 | -93 | 0 |

Author’s own work based on the Local Data Bank of *Statistics Poland*

economy to one based around the free market. This naturally had an unfavourable effect on many enterprises and industries. Indeed, a crisis ensued in the early 1990s, with unemployment soaring, and demands for transport services, both public and non-public, falling as a consequence. The detrimental effects did not spare the coach operator PKS – in essence a huge, unified organisation of nationwide reach, that now began being broken up into more than 170 autonomous enterprises (Taylor and Ciechański 2008).

For a start, that meant a major limiting of the services by which employees were transferred around the country, be these of a closed or open nature. That was in part symptomatic of the fact that large numbers of transport-enterprise (and of course many other) employees were losing their jobs, virtually from one day to the next. Collapsing state enterprises also engaged in a process of dispensing with their assets, be these large buses or coaches, or supply vehicles. Given the entrepreneurship coming naturally to Poles, the changes did bear fruit in the market appearances of dynamic new (smaller) enterprises posing both a theoretical and a real competitive threat to PKS. However, their means of operation were often specific, with bus-stops regularly being "cleared" of waiting people 5 minutes in advance of the timetabled arrival of PKS, by some rather-cheaper delivery-type vehicle.

Our study area began the transformation with five PKS enterprises seated within it. In the early 21$^{st}$ century, the carriers based in Gorlice, Sanok and Brzozów were subject to privatisation with the involvement of a foreign investor (the then Swedish firm *Connex*). Sadly, this process was not crowned by success as, while all the entities became consolidated into one, the successors of *Connex* (*Veolia Transport Polska* and *Arriva Bus Transport Polska*) withdrew steadily from servicing the area. December 2011 then saw the Gorlice branch of the firm (*Veolia Transport Bieszczady*) brought to liquidation as the first of the area's old PKS enterprises to go. When it came to the two others mentioned thus far (by then in the hands of *Arriva*), a complete cessation of carriage activity was ushered in on 30.06.2017 (Taylor and Ciechański 2020).

The stories with PKS Krosno and PKS Jasło were somewhat different. The former was commercialised in 2000 and became a single-person Treasury-owned company. However, the steady worsening of its condition necessitated another form of operation, so a process of communalisation ensued in 2011, with all shares passing into the hands of the poviat of Krosno authorities. However, the indebted firm came to represent too much of a burden for the county, which thus decided to sell an 85% stake to a financial-sector company called FK *Partner sp. z o.o.* And, notwithstanding supposed safeguarding provisions in the privatisation agreement, in late 2016 another complete cessation of carriage activity was instituted.

This left, as the sole active enterprise deriving from the PKS tradition, PKS Jasło (now PGZK *Jasiel*). This was worker-privatised, with the involvement of a strategic investor as well, in 2001. And as of 2018, the only shareholder was R. Rydarowicz, with the enterprise at that point finding itself in its peak condition. It was then in the summer of that year that PKS Jasło's restructuring gave rise to the PGZK *Jasiel* company. In May 2019, that enterprise was taken over by a *Poviat/Gmina Transport Association with its seat in Jasło County*.

The author of this research decided to start the analysis of the available data with the year 1990. On the one hand, this was the first year timetabled following the coming into being of a new economic reality for Poland. On the other, this was most likely the last year in which almost all of the bus or coach connections away from towns and cities were still being serviced by PKS enterprises, as still functioning on the basis of conditions that had pertained earlier in the centrally-planned economy. Still unchanged as of 1990 was the Gorlice–Jasło–Sanok–Lesko transport axis characterised not only by a very good bus/coach service, but also by a relatively good rail offer from PKP (Fig. 1). The attention is particularly drawn here to the Zagórz–Łupków section of line, reflecting the only circumstance in which the railway could be seen to play a visible, prevalent role. A kind of a curiosity at that time was a transit route via the USSR to Przemyśl that was served by one pair of local passenger trains and up to two pairs of long-haul services through to Warsaw.

The network of bus/coach connections was characterised in 1990 (Table 3) by a high degree of cohesion (for school days the cyclomatic number of the graph $\mu$=57, the Kansky index $\alpha$=11.56% and Kansky index $\gamma$=41.16%). A particular density of the system was achieved in the Gorlice, Jasło, Krosno and Sanok areas, which is to say in all the more major urban and industrial centres. From there, visible lines with heavy traffic are seen to run mainly in a

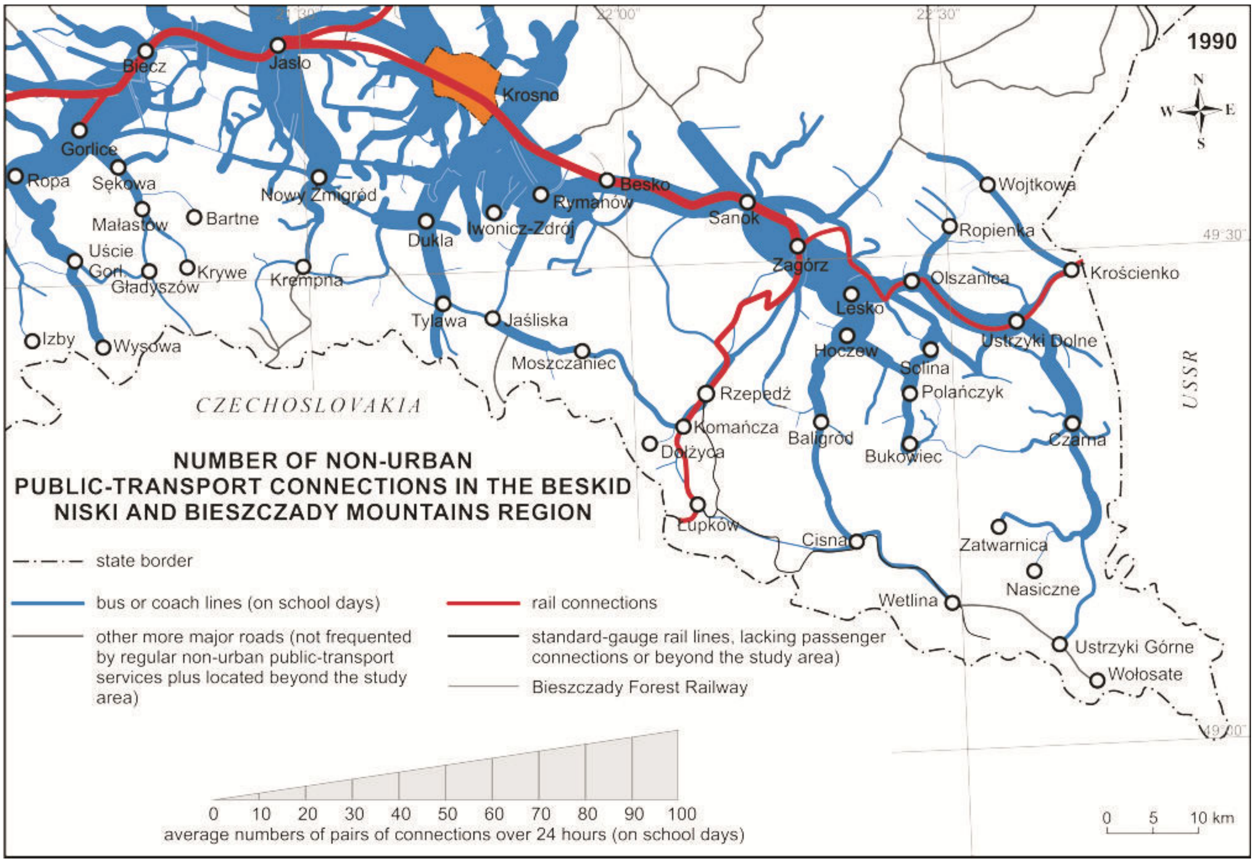

**Fig. 1** The passenger transport network in Poland's Beskid Niski and Bieszczady Mountain region in 1990. All figures in the article are the author's own elaboration.

**Table 3** Graph parameters and indices deriving from the analysis of non-urban bus-transport networks

| Year | Variants of network | Number of | | | Cyclomatic number $\mu$ | Kansky indices(%) | |
|---|---|---|---|---|---|---|---|
| | | graph edges, $e$ | graph vertices, $v$ | isolated subgraphs[a], $p$ | | $\alpha$ | $\gamma$ |
| 1990 | Working days | 305 | 249 | 1 | 57 | 11.56 | 41.16 |
| | Working days during summer holidays | 304 | 247 | 1 | 58 | 11.86 | 41.36 |
| | Non-working days | 265 | 229 | 1 | 37 | 8.17 | 38.91 |
| | Non-working days during summer holidays | 265 | 248 | 20 | 37 | 7.54 | 35.91 |
| 2019 | School days | 286 | 243 | 1 | 44 | 9.15 | 39.56 |
| | Working days | 270 | 243 | 17 | 44 | 9.15 | 37.34 |
| | Non-working days | 189 | 250 | 86 | 25 | 5.05 | 25.40 |
| | Non-working days during summer holidays | 189 | 249 | 86 | 26 | 5.27 | 25.51 |

**Note:** [a] – included isolated vertices. (Authors' own elaboration).

southerly direction. Such centres as Dukla and Nowy Żmigród were also very well-served by buses, as were the spas and health resorts of Iwonicz–Zdrój and Rymanów–Zdrój. Also characterised by heavy traffic were the stretches of road between Lesko and Ustrzyki Dolne, as stretching on to Czarna beyond. Quite noticeable is an uninterrupted parallel transport route leading from Gorlice to Wetlina (i.a. via Nowy Żmigród, Dukla and Cisna). In fact, such sections of route as Komańcza–Cisna or Gorlice–Nowy Żmigród characteristically had a rather limited offer where carriage was concerned. Also noteworthy was the way in which the Wetlina–Ustrzyki Górne stretch was only covered by a bus service in the summer-holiday period.

It should be noted how Jasło, Gorlice, Krosno and Sanok all had operating city-transport enterprises whose network of connections once extended far beyond the given set of town or city limits. Equally, certain large industrial plants at Gorlice and Jasło (as well as most probably Sanok and Krosno) organised their own closed services taking passenger traffic.

An interesting image of non-urban public transport is to be gained by taking account of the days on which means of transport ran (Fig. 2). What are noticeable are the two routes operating seasonally (one only in winter holidays and one only in summer holidays). In 1990, rail transport ensured its services through virtually the entire week. While the quality of the then network of connections was not the subject of analysis, a few localities in the Bieszczady and Beskid Niski chains remained deprived of weekend bus access. In the case of the Bieszczady Mountains, this was generally the case for just a couple of villages near Baligród, as well as places falling within the Sanocko–Turczańskie Mountains. In the Beskid Niski, areas continued to be deprived of weekend transport.

To sum up, as of 1990, the area of interest to us can be said to have had an effective system of public transport in place, with the achievement of transfers by both local people and tourists not presenting any more major obstacles at that time.

A period of less than 30 years proved sufficient to change the situation in the study area diametrically. Above all (as was noted above), for economic reasons, almost all the old PKS enterprises were subject to liquidation – with the sole exception being a company emerging from the PKS Jasło tradition. In exchange, in the Beskid Niski in particular, we now find several tens of smaller or larger private carriers in operation, mainly using delivery vehicles adapted to carry passengers, and only more rarely vehicles originally designated as buses.

The base of timetables brought together in the course of the research was updated in line with the early-2019 situation, with this then serving to devise maps showing on each single page the numbers and directions of connections achieved on a given average working day. There are also lines running only on working days or schooldays. No account was taken of closed runs organised by schools or employees at places of work, whose timetables never enter the public domain. The maps also show sections still serviced in 1990 but now bereft of mass public transport.

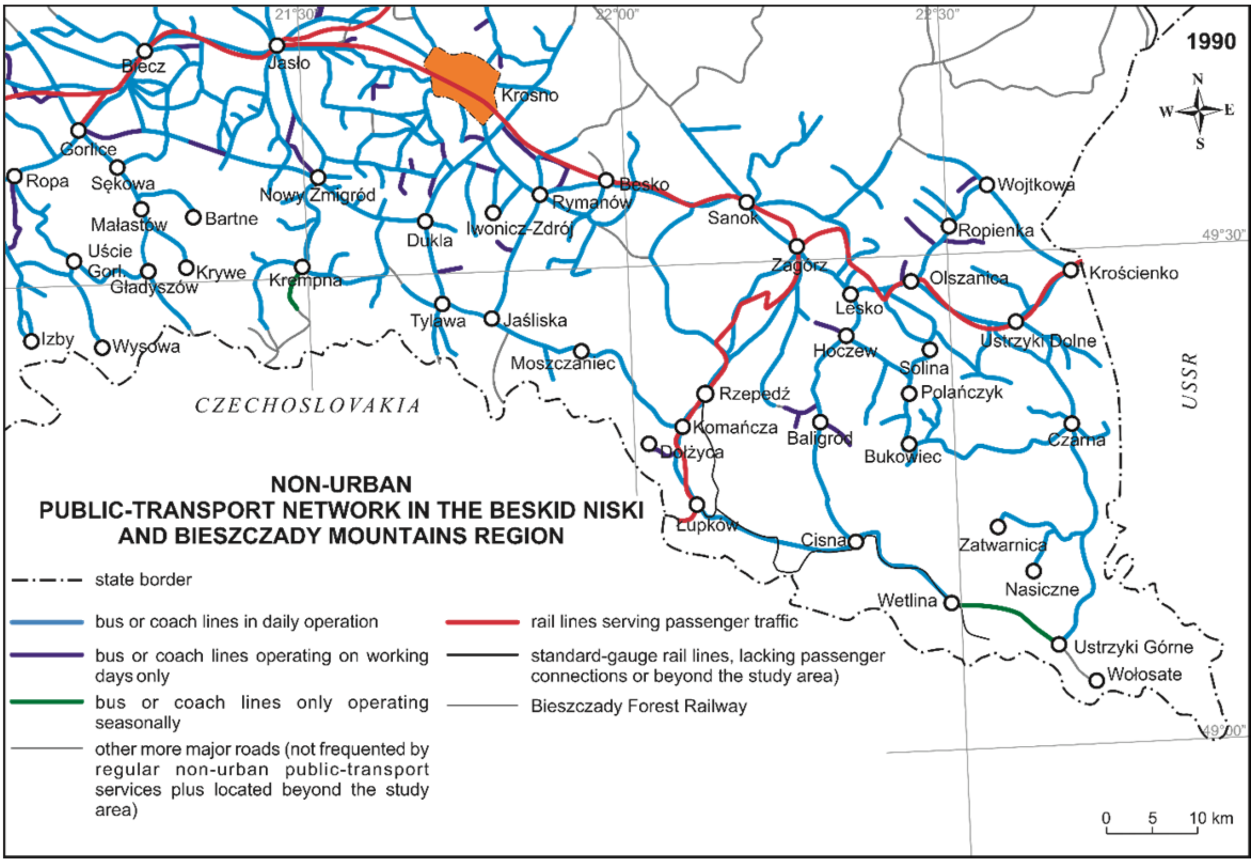

**Fig. 2** The passenger transport network in Poland's Beskid Niski and Bieszczady Mountain region in 1990, with a division into areas served daily or only on working days.

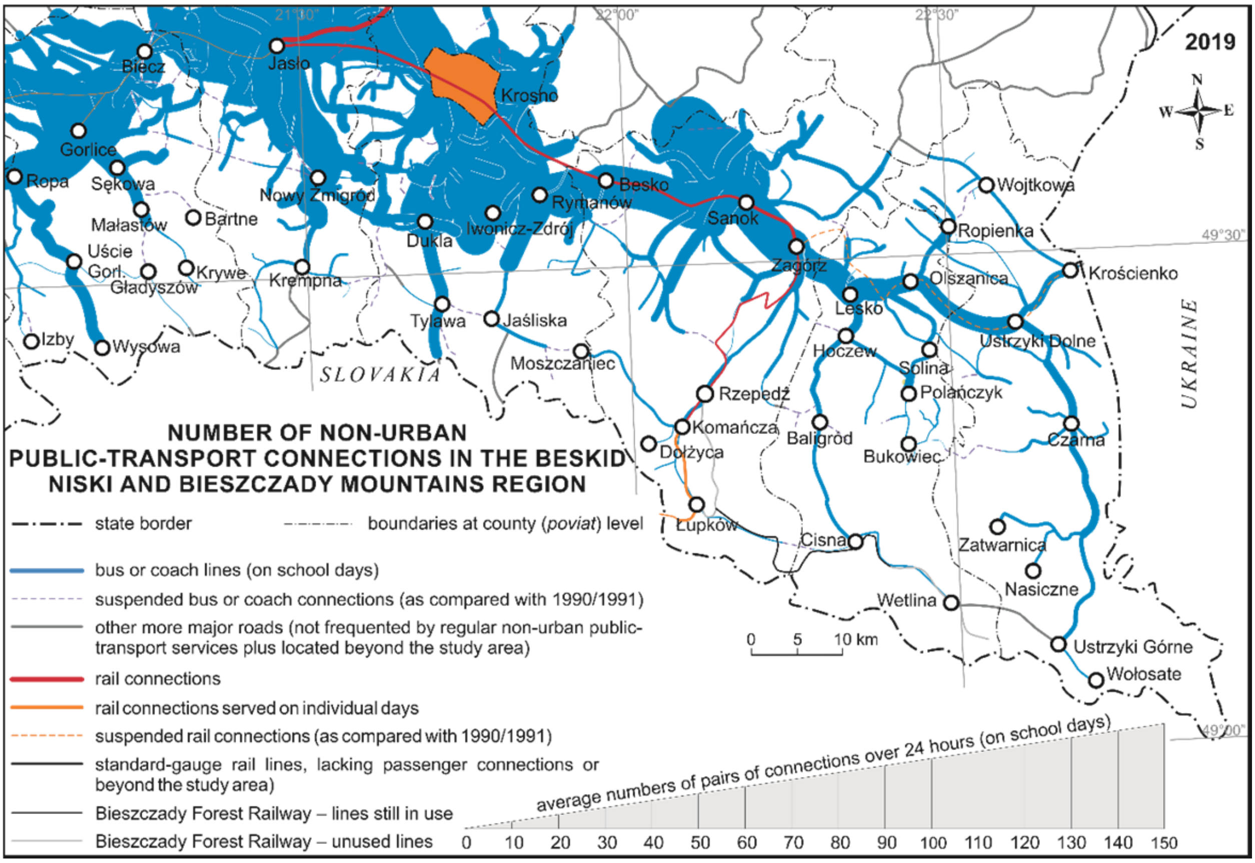

**Fig. 3** The passenger transport network in Poland's Beskid Niski and Bieszczady Mountain region in 2019.

The depiction in Fig. 3 is of the major collapse that the study area experienced between 1990 and 2019 where rail travel was concerned. A lack of renovation of infrastructure has unfortunately led to "technological death" and cessation of carriage along the line to Krościenko and on into Khyriv in Ukraine. A virtual cessation of operations (with occasional exceptions at weekends) has also taken place when it comes to carriage along the electrified line – still active in regard to goods traffic – between Stróże and Gorlice, Zagórzany and Jasło. The Jasło–Zagórz and Zagórz–Komańcza sections run a skeleton service (often transferring to a substitute railway-bus service). Equally, both the local line to Komańcza and the more significant Zagórz–Jasło line are inclined to suspend train traffic for periods every now and again, often with no substitute form of transport put forward in its place.

Where bus/coach transport in the years 1990-2019 is concerned, what is visible above all is growing polarisation of the network in comparison with the situation described for earlier periods. On the one hand, it is possible to note the increased numbers of buses travelling along the national route connecting Gorlice, Jasło, Krosno and Sanok; while on the other the southern, more-mountainous and less densely-populated parts of the studied poviats have mostly experienced a major deterioration in quality of what carriers have to offer. The loss of cohesion of the network should be a source of particular disquiet, while a new and unfavourable phenomenon is the lack of permeability of county boundaries. There is no way to reach Nowy Żmigród directly from Gorlice, Dukla from Krempna, Żubracze from Wola Michowa or Moszczaniec from Wola Niżna. Also ceasing to be possible is the bus journey from the Bukowiec area to Czarna, while the once-existing link between Tylawa and Jaśliska is also now lacking, even though both are in the same poviat. The same situation applies to Gorlice poviat, and its localities of Małastów and Gładyszów.

Values for the graph indices as presented in the previous section are seen to have changed. By 2019 (Table 3), the network of bus/coach connections was characterised by a high degree of cohesion (for school days a cyclomatic number of graph $\mu$=44, a Kansky index $\alpha$=9.15% and Kansky index $\gamma$=39.56%). But a decision to analyse connectivity of the network on non-working days reveals a more complicated situations, especially in the case of the cyclomatic number, which is down from $\mu$=38 in 1990 to $\mu$=26 in 2019 (and with a major increase in the number of isolated subgraphs from 20 to 86). On the one hand such a graph structure with a large number of isolated vertices is much more difficult to analyse from the topological point of view; but on the other hand, from a practical point of view, each such isolated point represents a place cut off from transport, where the lives of inhabitants are complicated to a marked extent.

The lack of connections is not the only problem observed here. The way in which the Solina Reservoir area is served has deteriorated markedly, while far fewer buses reach the gmina of Cisna out of season. A near-tragic situation applies to the south of Komańcza, as well as in the cases of some localities in the gminas of Uście Gorlickie, Krempna and Jaśliska. A similar level of service as in the past applies in the cases of Dukla, Nowy Żmigród and Wysowa. It is also possible to note unfortunately-rare processes of the return of services to a few villages, as well as facilitation of access from the Ropienka area to Ustrzyki Dolne.

The work did not involve a detailed analysis of the distribution of bus and coach runs throughout each 24-hour period. But it should be noted that the less-frequented routes are operated mainly in the rush-hours of traffic. The lack of direct connections is a further problem.

Unfortunately, the still-slightly-positive picture we might obtain from the research results discussed so far is spoilt irrevocably when we start to analyse traffic on non-working days (Fig. 4). It should first be recalled that – leaving aside the very few exceptions mentioned – localities back in 1990 could enjoy a full, seven-day, year-round public-transport service. Today's contrasting and dire situation is especially noticeable in the case of Lesko and Bieszczady Counties, where localities north of the National Road connecting Sanok and Krościenko lack access to public transport throughout the weekend. Likewise deprived are the areas around the Solina Reservoir, and some localities in the gmina of Lutowiska. The

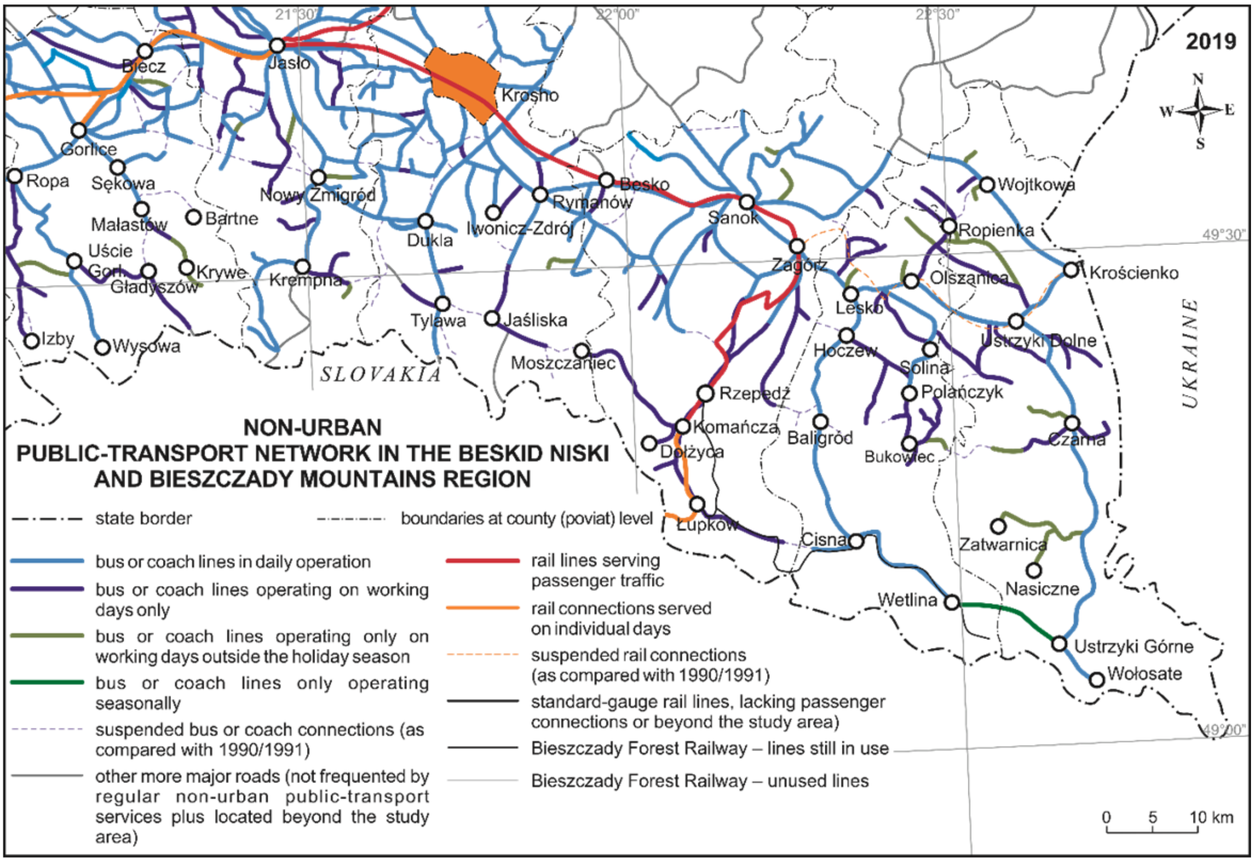

**Fig. 4** The passenger transport network in Poland's Beskid Niski and Bieszczady Mountain region in 2019, with a division into lines served daily, only on working days or only on schooldays.

situation in Sanok County offers little cause for optimism either, while the weekend transport "desert" also extends to many villages in the gmina of Komańcza. Were it not for the railways (or precisely the buses of substitute rail transport) running 3 times a day (just once on Sundays), Komańcza would likewise be cut off entirely from the world. Otherwise, Łupków was being serviced by trains on summer weekends only. Furthermore, no weekend coaches and buses were reaching the gmina of Jaśliska, while some villages in the southern part of the gmina of Dukla continue to fall victim to this kind of transport-based social exclusion. In Jasło County, a person might as well forget about making a weekend journey to some places in the gmina of Krempna. Within Gorlice County numerous localities in the Beskid Niski remain without weekend public transport (mainly in the gmina of Uście Gorlickie). Some of the routes around Biecz also lack services on these days.

It needs to be recalled how it is typical for remaining lines to be served by far fewer buses on Saturdays, while on Sundays and holidays many lines have just a single run – if there are any at all. An example here might be Dukla – serviced on working days by almost 70 trips run by three different carriers, but with this reducing to one on Sundays, with just 4 runs along the Dukla–Krosno route. However, on the days of the year (including Sundays) on which retailing is prohibited, no bus runs at all take place. Likewise, on Sundays, the route linking Krosno and Jasło is covered by express coaches and a few trains only.

A further new phenomenon observable now in comparison with the situation back in 1990 relates to the appearance of localities serviced on schooldays only (Fig. 4) – and hence a phenomenon that hinders the potential development of tourism. It can further be taken to signify that, during the school holidays, public transport does not exist in many villages of Bieszczady that are popular among tourists. Certain places in Beskid Niski go unserved, while the list of those deprived of public transport in summer extends further to include yet-more places.

## 5 Summary and Discussion

There is no doubt that 30 years of the transformation in Poland have brought about a major remodelling of the non-urban public-transport network in the area under study. First and foremost, there has unfortunately been a huge marginalisation where rail transport is concerned. At present, the local travel possibilities on offer (and the ways these are achieved) cast considerable doubt on the wisdom of these continuing to receiving any taxpayer support whatsoever.

Major losers 30 years into the economic and political transformation of Poland are then the enterprises once state-owned under the PKS umbrella. This has proved to be the case despite the various forms of ownership transformation enterprises were put through, given that only one such carrier has actually remained on the market, i.e. the one based in Jasło. The remainder of the enterprises in this category could not be saved – either by a process of privatisation that arrived too late, or by conversion to communal ownership.

Nevertheless, the biggest losers in this situation are the region's inhabitants. Even if numbers of connections have increased at first sight, it is very typical for scheduled journeys to have become confined to the most popular times of the day; or for what is on offer on non-working or non-school days to have declined very markedly in quantity and quality. These negative processes give rise to unfavourable consequences for particular people or groups thereof. A fundamental error appearing in publications on transport-based social exclusion is the way in which reference is made to cumulative values. For it is not numbers that have no way of returning home from work after 22.00; and it is not numbers, but especially children and young people – in the main from rural areas – that lack opportunities to participate in extra-curricular activities or schooling in the arts. And it is particular individuals – rather than numbers – that are unable to spend evenings with friends or take advantage of the cultural opportunities larger urban centres have to offer. It is also particular pupils and students unable to take up education in the place of their ideal choice, or in exactly the field they want; or else somehow condemned to seeing their families less often than they might.

There is thus a strong correspondence here with the conclusions set out in the report for Britain's Deputy Prime Minister and Prime Minister from the country's *Social Exclusion Unit* that was published in 2002 and 2003 under the title *Making the Connections*... Basically, poor transport is found to cause transport exclusion in two ways. The first involves limited access to the activities that increase people's life chances in fields such as work, education, healthcare, grocery shopping and other key activities. The second aspect has more of an environmental character.

Here it should be remembered that, although poor transport is only one of many factors causing social exclusion, it may be one of its key causes. Shergold and Parkhurst (2012) also rightly argued – where the South West of England and Wales were concerned – that rural housing and older age increase the risks of social exclusion. Taking into account the local demographic situation, similar processes should also be expected in the area of Poland under study here.

Unfortunately, therefore, these areas of Poland of a typically mountainous character have not benefited from 30 years of transformation in the country, as there are many localities reached by far fewer transport runs than was the case in 1990 (Fig. 5). Indeed, many places are cut off from public transport entirely, at least for periods of time; while others cannot be "escaped from" on non-working days or during the school-holiday period (approximately 2-3 months a year).

This would in turn coincide with the observations of Walsh et al. (2019) on the situation in Ireland and Northern Ireland – where access to public bus networks did not exist in several places by that time. Elsewhere (where public transport was available), the offer as regards connections was often weak and sporadic, with this deepening the impact of geographical distance and making it difficult for basic needs to be met. Although some programmes were considered to provide access to important purposes, they were again limited, for example with transport services only provided once a week in certain rural areas of Ireland.

What is more, the quality of the operation achieved by the non-urban public-transport system would seem to be very much in doubt. Fleets are typically of low quality, while attitudes to offers of carriage already announced look *ad hoc* at best, with timetables going unheeded, runs called off (and with no substitute service on offer), a non-transparent timetabling system by which to denote

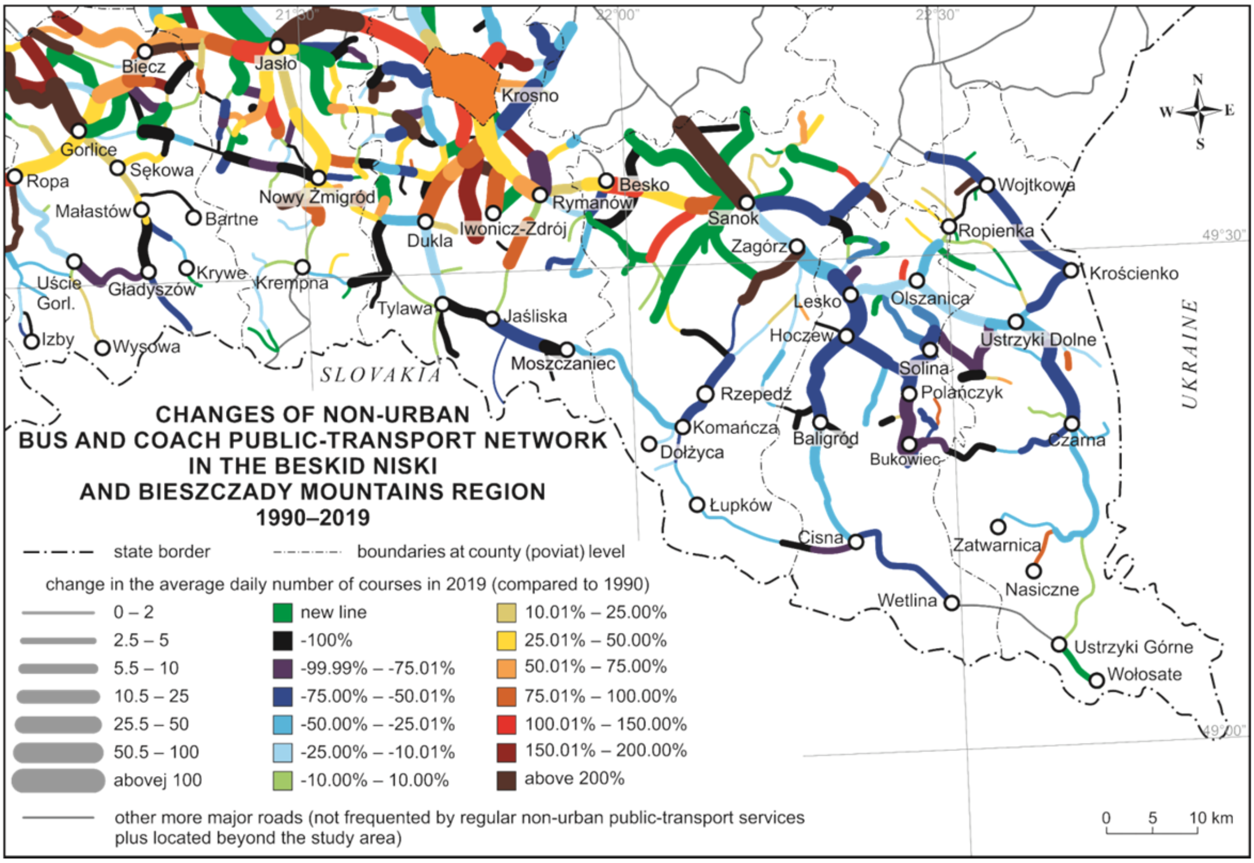

**Fig. 5** Change in the average daily number of bus connections of collective passenger transport on working days in the area of Poland's Beskid Niski and Bieszczady Mountains in 1990–2019.

times of journeys that are supposed to be made, and a rather "liberal" attitude on the part of many carriers to the apparent obligation to even make schedules public.

Of course one might always advocate private car ownership as a solution, or even one of the various forms of car-sharing; but it has to be recalled that this option will remain a difficult one for certain groups, or even out of reach – for reasons of age, finance, health or incurred risk (e.g. in line with a fear of assault that younger women may feel).

And ultimately a thought also needs to be given to tourists, whose access to the Beskid Niski and Bieszczady Mountains is obstructed. What is astounding is that public transport often disappears at the weekends in these areas. A paradoxical aspect is thus highlighted by the coaches running to Ustrzyki Górne via Wetlina – but only on working days. It can therefore come as no surprise that tourists to the Beskid Niski are left to their own devices, while in Bieszczady they take advantage of the buses run without a timetable or transport tariffs in season, while relying on the kindness of others in non-holiday periods of the year.

In recent months, the issue of transport-based social exclusion has become a particularly popular one in Poland's media, thanks to the government's introduction of a Fund for the Development of Coach Connections (*Fundusz Rozwoj Połączeń Autobusowych*). Unfortunately, it is easy to see how the public discourse on this subject includes a complete misunderstanding of the nature of the problem, which is often therefore distorted and blurred. First and foremost, it must be agreed that the actual idea of the Fund is a worthy and justified one in every sense – but just about 25 years too late! In the second place, it is hard to avoid the impression that those behind the idea did not fully see what the problem was, or how they wanted it to be resolved – a conclusion only reinforced when the lawmaking process is taken note of. And Dubicki (2019) is well able to confirm many of the author's observations regarding the issue of the said Fund, and in particular its weaknesses; and the many factors that earlier led to the collapse of public transport in Poland, as well as the consequences in the form of transport-based social exclusion.

However, in the view of the author, what was the major problem as the Fund made its appearance was the steady regression of the network of bus or coach connections – to the extent that the first designation of public funds should have sought to arrest that process. Only as a secondary objective would we arrive at any possible effort at rebuilding the network. Meanwhile, what actually happened was that the legislator did not leave local or regional authorities with any special freedom of choice, instead showing a clear preference for connections that had not existed at all for many months. Beyond that, it has been noteworthy that the sums allocated to top-up funding on a vehicle-kilometre basis do not suffice, while local authorities are not prepared to take up the slack. In any case, they lack a realistic needs analysis; while it is difficult for them to shift resources to expenditure not planned for in advance, and there is also a lack of the specialist personnel that would know enough about public-transport issues. For all these reasons, it can come as no surprise that it is not possible to refer here to any spectacular success of the government programme.

Unfortunately, the current crisis caused by the COVID-19 pandemic has only shown how fragile the financial foundations of public-transport operations involving coaches are; and how dependent on commutes to schools. Particularly worrying is the fact that, in areas of low population density such as those considered here, whole neighbouring counties (the *poviats* of Lesko and Bieszczady) faced a near-complete deprivation of access to public transport for two months (Ciechański 2020). This was not an isolated phenomenon, as – according to Oszter (2020) – the initial period of the pandemic in Ukraine, Slovenia and Croatia saw public transport suspended entirely. It was also pointed out that regions retaining reasonable possibilities as regards rail transport (albeit reduced by between 40 and 95% in the period), were still left in a better situation than many regions relying solely on bus transport (Taczanowski and Kołoś 2020; Smolarski 2020).

The alternative lack of support for non-urban bus transport is a further increase in the area of Poland and numbers of Poles subject to transport-based social exclusion. And this in the face of an ongoing process of ageing in society, ensuring the emergence of a simply massive social problem in coming years.

## Acknowledgments

The article was partly based on research within the framework of a project financed by the National Science Centre entitled: An empirical pilot study on transport exclusion in mountain areas based on the example of the Low Beskids and the Bieszczady counties (Miniatura 2, contract number 2018/02/X/HS4/00451).

**Open Access** This article is licensed under a Creative Commons Attribution 4.0 International License, which permits use, sharing, adaptation, distribution and reproduction in any medium or format, as long as you give appropriate credit to the original author(s) and the source, provide a link to the Creative Commons licence, and indicate if changes were made. The images or other third party material in this article are included in the article's Creative Commons licence, unless indicated otherwise in a credit line to the material. If material is not included in the article's Creative Commons licence and your intended use is not permitted by statutory regulation or exceeds the permitted use, you will need to obtain permission directly from the copyright holder. To view a copy of this licence, visit http://creativecommons.org/licenses/by/4.0/.

Funding: Open access funding provided by Polish Academy of Sciences.
